# T1-Weighted Intensity Increase After a Single Administration of a Linear Gadolinium-Based Contrast Agent in Multiple Sclerosis

**DOI:** 10.1007/s00062-020-00882-6

**Published:** 2020-02-13

**Authors:** S. Grahl, M. Bussas, V. Pongratz, J. S. Kirschke, C. Zimmer, A. Berthele, B. Hemmer, M. Mühlau

**Affiliations:** 1 Department of Neurology, Klinikum rechts der Isar, School of Medicine, Technical University of Munich, Ismaninger Str. 22, 81541 Munich, Germany; 2 TUM Neuroimaging Center, Klinikum rechts der Isar, School of Medicine, Technical University of Munich, Ismaninger Str. 22, 81541 Munich, Germany; 3 Department of Neuroradiology, Klinikum rechts der Isar, School of Medicine, Technical University of Munich, Ismaninger Str. 22, 81541 Munich, Germany; 4 Munich Cluster for Systems Neurology (SyNergy), Feodor-Lynen-Str. 17, 81377 Munich, Germany

**Keywords:** Brain MRI, Gadolinium deposition, Gadopentetic acid, Gadoteric acid

## Abstract

**Purpose:**

Through analysis of T1-weighted (T1w) images this study investigated gadolinium (Gd) deposition in the brain after administration of a linear (gadopentetic acid) and a cyclic (gadoteric acid) gadolinium-based contrast agent (GBCA) in patients with multiple sclerosis (MS), a disorder frequently requiring magnetic resonance imaging (MRI) scans over years.

**Methods:**

A total of 3233 T1w images (unenhanced with respect to the same scanning session) of 881 MS patients were retrospectively analyzed. After spatial normalization and intensity scaling using a sphere within the pons, differences of all pairs of subsequent scans were calculated and attributed to either linear (*n* = 2718) or cyclic (*n* = 385) or no GBCA (*n* = 130) according to the first scan. Regional analyses were performed, focusing on the dentate nucleus, and whole brain analyses. By 1‑sample t‑tests, signal intensity increases within conditions were searched for; conditions were compared by 2‑sample t‑tests. Furthermore, recent hypotheses on the reversibility of GBCA deposition were tested.

**Results:**

In the dentate nucleus, a significant increase was observed only after administration of linear GBCA even after a single GBCA administration. This increase differed significantly (*p* < 0.001) from the other conditions (cyclic and no GBCA). Whole brain analyses revealed T1w signal increases only after administration of linear GBCA within two regions, the dentate nucleus and globus pallidus. Additional analyses did not indicate any decline of Gd deposition in the brain.

**Conclusion:**

The data point towards Gd deposition in the brain after administration of linear GBCA even after a single administration.

## Introduction

Traditionally, gadolinium-based magnetic resonance imaging (MRI) contrast agents (GBCA) were considered to be the safest contrast agents with a very low rate of adverse events. This image was challenged by post-mortem histopathological studies [1, 2] demonstrating gadolinium (Gd) deposition in the brain after repeated GBCA administrations. This Gd deposition goes along with a measurable signal intensity increase in native T1-weighted (T1w) sequences [3]. Hence, Gd deposition is, to some degree, accessible to investigation in vivo, even longitudinally and even by analysis of T1w images derived in clinical routine. This study followed and adapted this approach with data from a large cohort of patients with multiple sclerosis (MS). Patients with MS may be especially vulnerable to potential long-term effects of Gd deposition in the brain as repeated MRI scans over many years are frequently indicated to monitor disease-modifying treatment. Furthermore, intervals between scans are mostly longer than in other populations that have been studied with respect to Gd deposition in the brain, such as patients with tumors [3–8]. Patients with MS may therefore show different kinetics and distribution of GBCA accumulation. Differences between a linear and a cyclic GBCA and kinetic aspects of Gd deposition were studied and increases of the T1w signal intensity were studied not only by region of interest analyses of the dentate nucleus area, where Gd deposition seems to be most robust [3, 7, 9–15] but also by whole brain analyses. Kinetic aspects of Gd deposition were also studied, in particular whether a certain number of GBCA administrations are needed for Gd deposition or whether a single administration suffices.

## Methods

### Participants

This retrospective single center study was approved by the internal review board and performed in accordance with the ethical standards laid down in the 1964 Declaration of Helsinki and its later amendments. Patients had given written informed consent for the use of their MRI data for research projects. The study included patients with MS (Table 1) and at least two MRI scans according to a standardized protocol between 2009 and 2017. Exclusion criteria were comorbidities possibly interfering with image analysis, lesions within the control region for scaling and further GBCA administrations between pairs of scans (according to the hospital information system). In 2015, the standard changed from the linear GBCA gadopentetic acid (Magnevist®, Bayer Vital GmbH, Leverkusen, Germany) to the cyclic GBCA gadoterate meglumine (Dotarem®, Guerbet group, Roissy, France). Of the 881 patients included, 428 received both linear and cyclic, 395 exclusively linear, 53 exclusively cyclic and 5 no GBCA. Standard dosage was used (0.1 mmol/kg body weight, up to 10 mmol total).

**Table 1:** Summary of demographic and clinical data

		Linear GBCA	Cyclic GBCA	No GBCA (control)	All conditions 1st scan	All conditions last scan
*n*		2718	385	130	3233	3233
Mean age years (SD)		37.5 (10.4)	39.1 (10.6)	37.7 (9.8)	36.2 (10.6)	39.8 (10.9)
Sex (% female)		66.7	68.1	63.8	68.0	68.0
Mean EDSS (SD)		1.7 (1.7)	1.5 (1.5)	1.3 (1.2)	1.6 (1.7)	1.6 (1.7)
Mean disease duration in years (SD)		3.7 (4.3)	5.3 (5.0)	5.1 (4.5)	2.1 (4.6)	5.8 (5.3)
Mean number of administrations (SD)		5.4 (4.0)	1.4 (0.7)	–	–	–
Diagnosis in %	CIS	–	–	–	28.9	11.8
	RRMS	–	–	–	66.8	83.3
	SPMS	–	–	–	2.8	3.5
	PPMS	–	–	–	1.5	1.5

*n* number, *SD* standard deviation, *CIS* clinically isolated syndrome, *EDSS* expanded disability status scale, *GBCA* gadolinium-based contrast agent, *n* number, *MS* multiple sclerosis, *RRMS* relapsing-remitting MS, *SPMS* secondary progressive MS, *PPMS* primary progressive MS

### Image Acquisition and Processing

**Fig. 1:**
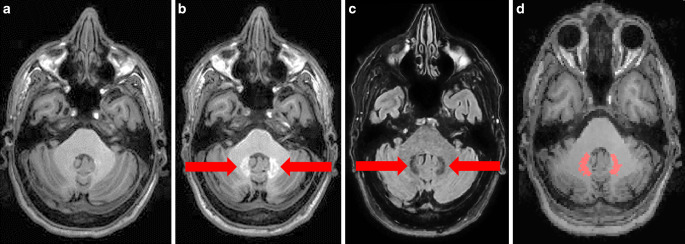
A patient with visible T1w signal intensity increase within the dentate nucleus is shown. **a** N_linear GBCA_ = 0, N_linear GBCA_ = 19, **c** FLAIR image, **d** dentate nucleus mask. Native T1w images were acquired in 2009 (**a**) and after 20 applications of linear gadolinium-based contrast agents (GBCA), in 2016 (**b**); the dentate nucleus appears brighter (*arrows*); from the FLAIR image (**c**) and the dentate nucleus mask of FSL SUIT toolbox (not shown), the customized dentate nucleus mask was generated and projected on the normalized T1w image (**d**, *mask in red*)

**Fig. 2:**
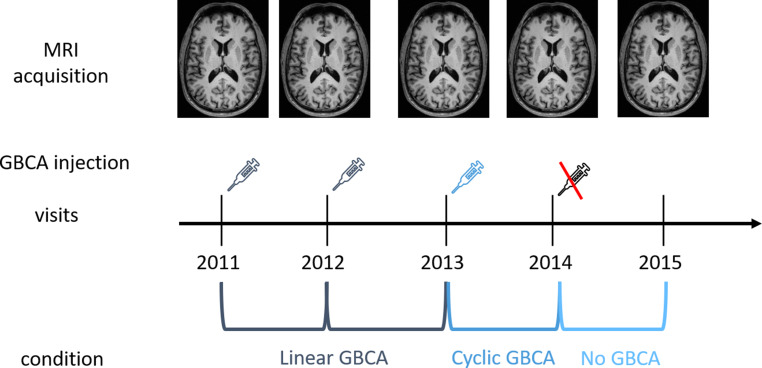
The study design is illustrated by an exemplary patient. Gadolinium-based contrast agent (GBCA) was applied in 2011, 2012 (both linear GBCA), and 2013 (cyclic GBCA) but not in 2014. This patient contributes difference images between two subsequent timepoints to the linear GBCA condition (2011/2012; 2012/2013), the cyclic GBCA condition (2013/2014) and the control (i.e. no GBCA) condition (2014/2015)

High resolution MRI was performed (3 T, Achieva, Philips, Best, The Netherlands) and 3D spoiled gradient echo T1w sequences were applied with the following parameters: voxel size = 1 mm isotropic, TR = 9 ms, TE = 4 ms. Further turbo-spin echo T2w FLAIR (fluid attenuation inversion recovery) images were acquired with the following parameters: voxel size = 1.0 × 1.0 × 1.5 mm; TR = 10,000 ms; TE = 140 ms; TI = 2750 ms. All images were preprocessed and normalized with SPM12 and its toolboxes CAT (http://www.neuro.uni-jena.de/cat/index.html) and LST (lesion segmentation tool) 2.0.15 (http://www.statistical-modelling.de/lst.html) with the default options resulting in T1w images, which are bias-corrected, normalized to MNI (Montreal Neurological Institute) space, and with the white matter lesions filled with intensities of normal appearing white matter as described earlier [16]. Next, the region of interest (dentate nucleus) and the control region (pons) were defined. The dentate nucleus mask of FSL’s (FMRIB Software Library; FMRIB, Oxford Centre for Functional MRI of the Brain) SUIT (spatially unbiased atlas template of the cerebellum and brainstem) toolbox was projected onto individual FLAIR (fluid attenuation inversion recovery) images (normalized to MNI space after coregistration to the T1w images), since the contrast of the dentate nucleus is higher in FLAIR sequences (hypointense) than in T1w images. Median FLAIR intensity of the dentate nucleus region was calculated and only voxels with lower intensity than the median intensity (within the SUIT dentate nucleus mask) built the final individual dentate nucleus mask (Fig. 1). For scaling, median signal intensity within the control region of the pons was determined in a sphere of 5 mm in diameter around the MNI coordinate x = 0, y = −26, z = −36. The FLAIR images were visually checked for white matter lesions in the dentate nucleus or pons region. In cases of white matter lesions in these regions, images were excluded (*n* = 10) because white matter lesions lead to signal changes, not intended to be measured here. Individual dentate nucleus and pons masks were projected onto the unenhanced T1w image in MNI space, median voxel intensity was calculated for the respective regions and the DN-to-P (dentate nucleus-to-pons) ratio (DN/P) was built. For regional analyses, DN/P values of two subsequent time points were subtracted (later minus earlier image). Resulting difference values were attributed to one of the three conditions (linear, cyclic or, as control no GBCA). Fig. 2 depicts an exemplary assignment of images of one patient to either of the three conditions. In total 2718 difference values were included in the linear condition, 385 in the cyclic condition and 130 in the control condition. For whole brain analyses, the normalized and bias-corrected T1w images were calibrated by dividing voxel intensity values by the median pons intensity value. Then, difference images were calculated by subtracting the calibrated T1w images from two subsequent time points. Difference images were also attributed to one of the three conditions (linear, cyclic or control).

### Statistical Analysis

A total of three types of analyses were performed. First, the potential influence of several parameters on the T1w signal changes in the dentate nucleus (of the difference images), such as number of previous GBCA administrations (i.e. testing for non-linear effects), age, and sex were explored. Searching for GBCA-related changes of the MRI signal in the control region pons that could disturb the analyses, the median pons intensity was plotted over the number of GBCA administrations separately for linear and cyclic GBCA. We further assessed the relationship between latest EDSS score and maximum number of GBCA administrations per person. Second, we focused on the dentate nucleus as increases in intensity after GBCA administration in this regions had been consistently reported [3, 7, 9–15]. The software package R (Version 3.2.3, Rstudio, Boston, MA, USA) was used and *p*-values <0.05 were considered statistically significant. By one-sample t‑tests within each condition, we tested whether mean signal intensity changes were significantly larger than zero. By two-sample t‑tests, DN/P differences were compared between conditions. We also tested recent hypotheses that GBCA deposition in the brain is reversible to some degree including the observation of a washout of linear GBCA when followed by the administration of cyclic GBCA [4, 17]. Therefore, we additionally analyzed two subconditions of the conditions cyclic GBCA and no GBCA, including only difference images of subjects having received linear GBCA beforehand. This resulted in the subconditions cyclic GBCA after linear GBCA and no GBCA after linear GBCA. For example, the cyclic condition illustrated in Fig. 2 contributed to the subsample cyclic after linear GBCA as the second linear GBCA condition preceded the cyclic GBCA condition. Third, we performed whole brain analyses to search for further areas with an increase of the T1w signal after GBCA administration. Whole brain voxel-based analysis was performed in SPM12 using the T1w difference images after smoothing with a Gaussian kernel of 4 mm full width at half maximum. Again, we performed one-sample t‑tests against zero for each of the conditions and two-sample t‑tests to compare conditions. Voxel-wise *p*-values <0.05, after family-wise error correction across the whole brain, were considered statistically significant.

## Results

**Fig. 3:**
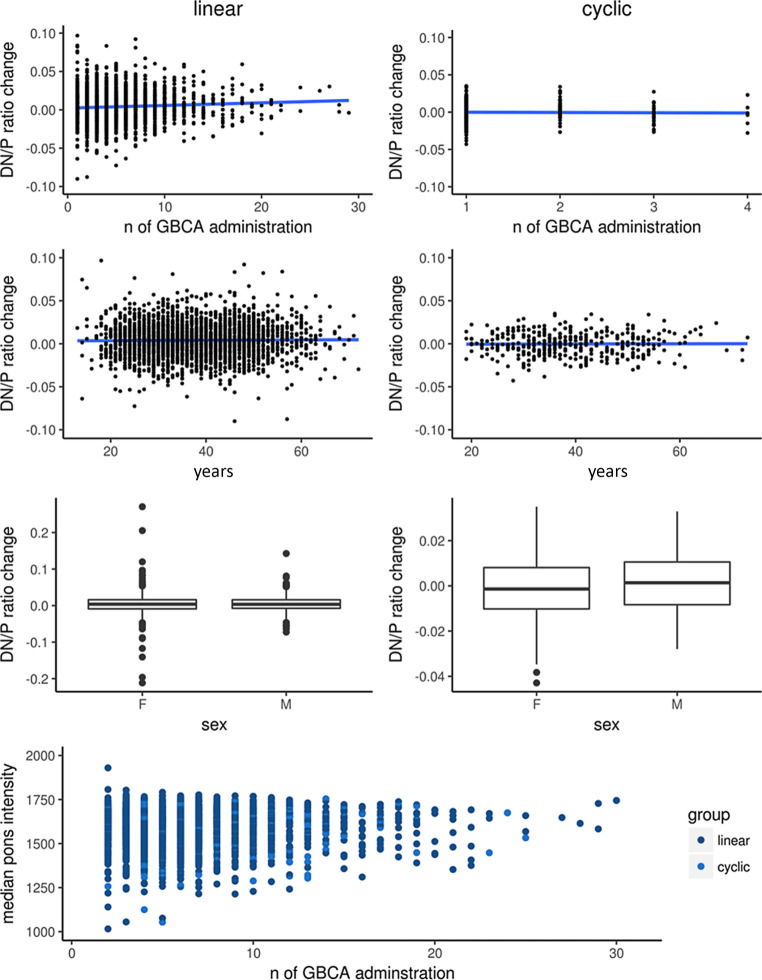
Parameters potentially interfering with signal intensity changes after application of gadolinium-based contrast agent (GBCA) were analyzed. In the upper three panels, changes (per pair of consecutive images) in the intensity ratio of the dentate nucleus (DN) and pons (P) after application of linear (*left column*) and cyclic (*right column*) GBCA are plotted against the number of applications, age, and sex; Pearson correlation of signal change and number of previous GBCA administrations in the linear condition was significant, but very small (R2 = 0.004), no systematic influence was observed for the cyclic condition as well as for age and sex in both conditions. In the lower panel, the intensity values of the control region (pons) is plotted against number of previous GBCA administration without an indication of a systematic shift (linear GBCA in *dark blue*, cyclic GBCA in *light blue*) of the T1w signal over time (R2 = 0.0004). *n* number, *F* female, *M* male

We did not find any indications that any of the potentially confounding parameters interfered with the signal intensity changes after administration of GBCA in a meaningful way (Fig. 3). In particular, the signal change between subsequent scans was almost independent of the number of GBCA administrations beforehand. These analyses for non-linear effects (Fig. 3, upper panel) showed a significant increase (linear: R2 = 0.04; *p* = 0.001; cyclic: R2 = 0.0004; *p* = 0.7) of the signal changes after linear GBCA. Yet, this effect was very small explaining only 0.4 per mille of the variance of signal increase which justified our approach to analyze pairs of subsequent images (i.e. difference images). Further, neither age nor sex showed an effect on the signal intensity changes after administration of GBCA (Fig. 3, middle panels). Moreover, the T1w signal of the control region pons varied but did not systematically change with respect to the number of previous GBCA administrations (Fig. 3, lower panel, R2 = 0.0001, *p* = 0.5). As expected, EDSS score correlated with maximum number of GBCA administration per subject (R2 = 0.01, *p* = 0.005).

**Fig. 4:**
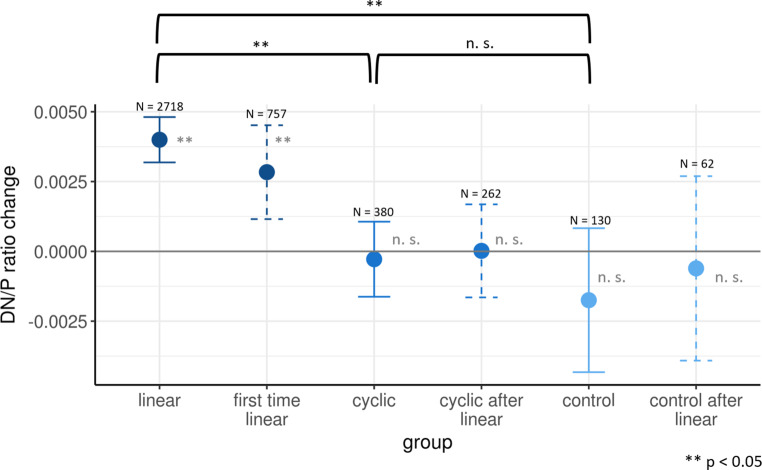
Mean values and 95% confidence intervals of T1w signal changes within the dentate nucleus are shown. One-sample t‑tests (significance indicated by *grey asterisks*) indicate a signal increase only after application of linear gadolinium-based contrast agents (GBCA), already after the first application (“first time linear”). The two sample t‑tests for the three main conditions (significance indicated by *black asterisks*) indicate differences in T1w signal intensity changes after application of linear GBCA compared to cyclic GBCA and no GBCA (control). Analyses of subgroups within the cyclic and control condition (previous administration of linear GBCA) do not suggest washout effects. *First time linear* linear GBCA administered for the first time; *cyclic after linear* cyclic GBCA condition follows linear GBCA condition; *control after linear,* control condition follows linear condition; *n.* *s.* not significant; *N* number of scans per analysis

The regional analyses of the dentate nucleus region through one-sample t‑tests revealed a significant increase of the DN/P ratio only after administration of linear GBCA (Fig. 4). In extreme cases, this could be detected visually (Fig. 1). Of note, when focusing on the other end of the spectrum, a DN/P ratio increase could also be demonstrated by summing up only the difference images after first time administration of linear GBCA (Fig. 4). When comparing the conditions of different GBCA directly by two-sample tests, we found significantly higher increases of the DN/P ratio after administration of linear GBCA compared to cyclic and no GBCA (Fig. 4). Subcondition analyses (cyclic GBCA condition following linear GBCA condition, control condition following linear GBCA condition) did not suggest a decrease of the DN/P ratio and, hence, any washout of linear GBCA (Fig. 4).

**Fig. 5:**
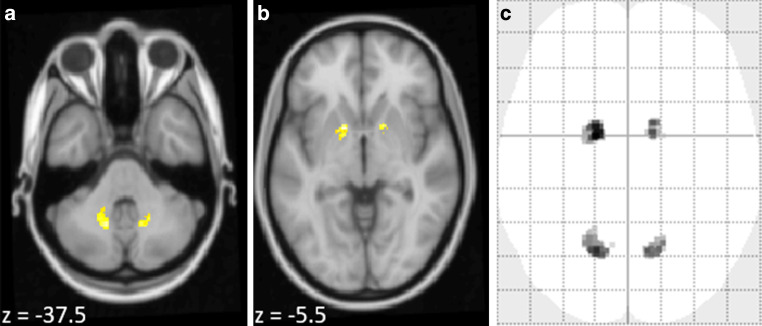
Whole brain voxel-based analysis of T1w signal increase after administration of the linear gadolinium-based contrast agent gadopentetic acid is shown. The one-sample t‑test of difference images reveals an increase in the T1w signal not only in the dentate nucleus but also in the globus pallidus (family-wise error corrected, p < 0.05). Significant voxels are projected on the T1w template of SPM with MNI coordinates of the z‑axis given in the lower left corners (**a**, **b**). Maximum intensity projection indicates that no further area was identified (**c**)

Our whole brain analyses showed significant T1w signal increases only after administration of linear GBCA. Besides the dentate nucleus, we observed T1w signal increase bilaterally in the globus pallidus (Fig. 5), which is well in accordance with other studies [1–3, 7, 12, 13, 18, 19]. Comparison between conditions did not demonstrate significant differences after correction for multiple comparisons across the whole brain.

## Discussion

We studied T1w signal intensity changes after administration of GBCA in MS patients. We found intensity increases, suggesting Gd deposition, in the dentate nucleus and pallidum only after administration of the linear GBCA gadopentetic acid. Of note, we demonstrated this signal increase already after a single administration of linear GBCA. Our finding of T1w-signal increase after administration of the linear but not the cyclic GBCA is in line with most [5, 6, 8, 11, 20] but not all [12, 19] in vivo human MRI studies. Likewise, some [21, 22] but not all [18, 23] animal studies demonstrated Gd deposition in the brain after repetitive administration of cyclic GBCA as well. Further in one post-mortem study, deposits of Gd after administration of two different cyclic GBCA (gadoteridol, gadobutrol) were demonstrated; yet, the authors speculated that Gd may have been deposited in its chelated form [24]. This assumption is well in line with a later animal study on the cyclic GBCA gadoteric acid; in which only traces of the intact chelated gadolinium were observed in the brain. Of note, these chelated Gd deposits followed a time dependent clearance [25], suggesting the need to differentiate between chelated and dechelated Gd [26].

With this in mind, our results point to a higher concentration of Gd deposits in the brain after administration of linear compared to cyclic GBCA but do not exclude Gd deposition after administration of cyclic GBCA.

To the best of our knowledge, our analytical approach, based on difference images per GBCA administration, has not yet been used in studies on Gd deposition in the brain. We believe that by including all available images instead of only the first and last images of each subject, we could leverage more information from our data. Given the only small T1w signal increase after GBCA administration, averaging was nevertheless necessary to detect meaningful signals. With our approach, we did not primarily average across multiple GBCA applications per subject, as done in other studies on Gd deposition in the brain [3–8, 11, 14, 15], but across subjects per single GBCA administration. In this way, single subjects contributed data to more than one condition (linear, cyclic, no GBCA), which was important as our study was done retrospectively in a cohort over a period of time in which routine administration of GBCA switched from the linear GBCA gadopentetic acid (Magnevist®) to the cyclic GBCA gadoterate meglumine (Dotarem®). This particular approach allowed us to address questions with respect to the kinetics of Gd deposition. Are several administrations necessary to cause considerable Gd deposits in the brain? This is, for example, suggested by the FDA Drug Safety Communication of 27 July 2015 entitled “FDA evaluating the risk of brain deposits with repeated use of gadolinium-based contrast agents for magnetic resonance imaging (MRI) [27, 28].” In this document, it is stated that “deposits of GBCAs remain in the brains of some patients who undergo four or more contrast MRI scans.” This statement is also compatible with the notion that biological meaningful non-linear effects come into play so that exceeding a certain number of administrations must be regarded particularly critical as also suggested by one study on T1w signal increase after multiple GBCA administrations [5]. Alternatively, Gd deposition may be an incremental (i.e. linear) process during which the detection threshold is exceeded after a certain number of GBCA administrations, which is supported by a post-mortem study on Gd accumulation after GBCA administration [29]. Of note, the number of previous GBCA applications exerted a significant effect: the more times GBCA had been administered, the higher the increase per single GBCA administration; however, this non-linear component of the effect was very small, explaining only four per mille of the variance of the observed T1w signal increase. Based on the assumption of linearity, we could demonstrate an increase of the T1w signal, and hence probable Gd deposition, after only one GBCA administration. Our approach also enabled to address the issue of a possible Gd washout by investigating subconditions with respect to a decrease of the T1w signal. In contrast to previous studies, we did not find any indication of a washout of Gd deposits neither over time [25, 26] nor when linear GBCA were followed by cyclic GBCA [13, 14, 29, 30]. Alternatively, Gd washout may follow kinetics not detectable by our study, in which relatively homogenous intervals (about 11 months) were analyzed. Taken together, we believe that accumulation of Gd deposition after linear GBCA follows a function of superimposed rectangular signals and that potential risks of linear GBCA applications are likely to be incremental challenging the idea of a safe threshold with respect to the number of GBCA administrations.

No robust indication exists so far that Gd deposition after GBCA administration goes along with side effects. Yet those could, for example by accelerating neurodegeneration, come into play years or even decades after exposition. Therefore, the potential risks of patients with MS, who frequently undergo regular follow-up scans over years to monitor therapy, seem to be particularly considerable. We believe that the significant correlation between EDSS score and number of GBCA administrations is of little value in this respect. On the contrary, we expected this association, assuming that patients with more severe courses (including highly active patients) are more likely to be scanned more frequently and, hence, to receive more GBCA administrations. Of note, another study investigated the sodium-related MRI signal, as a surrogate marker of potential tissue damage, in MS patients. Within areas of increased T1w signal intensities of the DN, a normal sodium signal was determined and, hence, no evidence of GBCA-related tissue damage provided [30]. In addition, there may be kinetic aspects to be considered for the evaluation of MS patients with respect to Gd deposition. In our study, the mean interval between two GBCA administrations (11 months) was longer than in studies on patients with tumors, for example 3 months [7]. We speculated that longer intervals go along with less accumulation. Yet, this does not seem to be the case. In line with four other studies [11, 14, 15, 20], patients with MS do not show different T1w signal increases than tumor patients.

Our MRI data comprised exclusively data from a single standardized protocol including FLAIR and T1w 3D sequences. This let us to undertake a voxel-wise whole brain analysis of T1w signal increase. In accordance with other studies applying region of interest analyses, we identified the dentate nucleus and globus pallidum indicating most Gd deposition in these brain regions. This approach has two main assumptions. First, the control region for scaling, the pons in our case, must not show the effect of interest. Some histopathological studies found Gd retention in the pons region although in a much lower concentration compared to the dentate nucleus [2, 24, 29]. Hence, deposition of lower Gd concentrations in other brain areas (than the pallidum and dentate nucleus) cannot be ruled out. Second, the correction of the bias field needs to be precise, which is not directly measurable. Yet our result of T1w signal increase within the pallidum, centimeters away from the control region for scaling and reported to be a region of pronounced Gd deposition [1–3, 7, 8, 12, 13, 15, 19, 29], suggests that our bias correction performed well.

We acknowledge limitations of our study. The number of observations was larger in the condition linear GBCA compared to the conditions cyclic or no GBCA. Yet the number of difference images after the first administration of linear GBCA was of the same magnitude as that of cyclic GBCA and we still demonstrated a significant increase of the T1w signal. Moreover, the number of 380 difference images after cyclic GBCA was still relatively high. We also acknowledge that the results of our subcondition analyses are based on relatively low numbers of observations; nevertheless, a trend pointing towards the hypothesized effect was not observed in any of the subconditions.

In conclusion, our findings suggest that administration of linear GBCA leads to Gd deposition in the globus pallidum and, already after a single administration, in the dentate nucleus.
